# A meta-analysis of MRI radiomics-based diagnosis for BI-RADS 4 breast lesions

**DOI:** 10.1007/s00432-024-05697-3

**Published:** 2024-05-15

**Authors:** Jie Lin, Hao Zheng, Qiyu Jia, Jingjing Shi, Shiwei Wang, Junna Wang, Min Ge

**Affiliations:** 1 The First Affiliated Hospital of Zhejiang Chinese Medical University (Zhejiang Provincial Hospital of Chinese Medicine), Hangzhou, China; 2 The First Affiliated Hospital of Xinjiang Medical University, Xinjiang, China

**Keywords:** BI-RADS 4, MRI, Radiomics, Breast lesions, Breast cancer

## Abstract

**Objective:**

The aim of this study is to conduct a systematic evaluation of the diagnostic efficacy of Breast Imaging Reporting and Data System (BI-RADS) 4 benign and malignant breast lesions using magnetic resonance imaging (MRI) radiomics.

**Methods:**

A systematic search identified relevant studies. Eligible studies were screened, assessed for quality, and analyzed for diagnostic accuracy. Subgroup and sensitivity analyses explored heterogeneity, while publication bias, clinical relevance and threshold effect were evaluated.

**Results:**

This study analyzed a total of 11 studies involving 1,915 lesions in 1,893 patients with BI-RADS 4 classification. The results showed that the combined sensitivity and specificity of MRI radiomics for diagnosing BI-RADS 4 lesions were 0.88 (95% CI 0.83–0.92) and 0.79 (95% CI 0.72–0.84). The positive likelihood ratio (PLR), negative likelihood ratio (NLR), and diagnostic odds ratio (DOR) were 4.2 (95% CI 3.1–5.7), 0.15 (95% CI: 0.10–0.22), and 29.0 (95% CI 15–55). The summary receiver operating characteristic (SROC) analysis yielded an area under the curve (AUC) of 0.90 (95% CI 0.87–0.92), indicating good diagnostic performance. The study found no significant threshold effect or publication bias, and heterogeneity among studies was attributed to various factors like feature selection algorithm, radiomics algorithms, etc. Overall, the results suggest that MRI radiomics has the potential to improve the diagnostic accuracy of BI-RADS 4 lesions and enhance patient outcomes.

**Conclusion:**

MRI-based radiomics is highly effective in diagnosing BI-RADS 4 benign and malignant breast lesions, enabling improving patients’ medical outcomes and quality of life.

## Introduction

Breast cancer (BC) remains a prevalent malignancy worldwide, accounting for approximately 31% of all cancer cases in women, with a mortality rate showing an annual increase of 415 (Siegel et al. 2023). In line with the breast cancer screening guidelines provided by the National Comprehensive Cancer Network (NCCN) and the Breast Imaging Reporting and Data System (BI-RADS), suspicious breast lesions are typically classified into six distinct categories (Goetz et al. 2019). Among these categories, BI-RADS 4 breast lesions are characterized as being suspicious for malignancy with an unknown pathological type, presenting a wide range of malignancy probabilities ranging from 2 to 95% (including 4a:2–10%,4b:10–50%,4c:50–95%). To further investigate and establish an accurate diagnosis, needle biopsy is commonly performed as a diagnostic procedure (Bennani-Baiti et al. 2017). However, the absence of clear qualitative characteristics for BI-RADS 4 breast lesions has led to overdiagnosis and unnecessary needle biopsies, causing physical harm to patients. Like any clinical diagnostic method, there is a chance of false positives and missed diagnoses (Gradishar et al. 2017). Therefore, the development of noninvasive and efficient diagnostic methods for breast BI-RADS 4 lesions holds great significance.

Breast magnetic resonance imaging (MRI) has emerged as a crucial tool for evaluating and detecting breast lesions. In routine clinical examinations, it is widely employed alongside mammography and ultrasonography to diagnose breast cancer. It provides vital information regarding tumor size, location, and invasion extent, aiding in the assessment of lymph node and tissue involvement. (Sohn and Bisdas 2020). Radiomics is an emerging approach that involves extracting high-throughput image features from medical images and conducting quantitative analysis. This is done by annotating abnormal findings and subsequently applying routine image processing techniques, which helps in obtaining diagnostic outcomes. Due to its capacity to enhance the diagnostic accuracy of radiologists, radiomics has garnered significant attention in the field of diagnostic image analysis (Ye et al. 2020). While there is significant research backing the use of MRI radiomics in diagnosing benign and malignant breast lesions (Satake et al. 2022), previous studies examining the effectiveness of MRI radiomics in diagnosing BI-RADS 4 lesions have been hindered by small sample sizes, limited statistical analyses, and inconsistent research findings. Therefore, a comprehensive meta-analysis is warranted to assess the diagnostic efficacy of MRI radiomics in differentiating between benign and malignant breast lesions categorized as BI-RADS 4.

## Materials and methods

The meta-analysis was conducted following the guidelines outlined in the Preferred Reporting Items for Systematic Reviews and Meta-Analyses (PRISMA) statement (McInnes et al. 2019).

### Literature search

In this study, LJ and ZH conducted a systematic search across four publicly available databases: PubMed, Embase, Cochrane Library, and Sinomed. The search included studies published until May 17, 2023, employing a Boolean algorithm with the following keywords: ((machine learning OR texture OR artificial intelligence OR radiomics) AND (((magnetic resonance OR MRI OR MRI Scan OR Magnetic Resonance Imaging OR Magnetic Resonance Tomography)) AND ((Breast Lesion OR Breast Tumor OR suspicious breast lesion OR breast mass)) AND ((Breast Imaging Reporting and Data System OR Breast Imaging Reporting and Data System score OR BI-RADS)). The reviewers then independently evaluated the titles and abstracts of the identified articles to determine their eligibility. To ensure the quality of the research, we excluded case reports, nonoriginal investigations (such as editorials, letters, and reviews), and studies that were not relevant to the research topic. In cases where there were differences in opinion between the two reviewers, we they resolved them through constructive discussion and came to a consensus.

### Literature inclusion and exclusion criteria

Two reviewers, adhering to the PICO (Population, Intervention, Comparison, Outcome) principles, developed specific inclusion and exclusion criteria to identify eligible studies for inclusion in the meta-analysis. Inclusion criteria are as follow: (1) The literature included subjects with breast lesions diagnosed as either benign or malignant based on pathological criteria. (2) The subjects underwent breast MRI examination prior to undergoing biopsy or excision surgery. (3) All subjects in the literature were diagnosed with BI-RADS 4 breast lesions following MRI examination. (4) The studies involved radiomics analysis conducted on breast MRI images. Exclusion Criteria are as follow: (1) The subjects included in the study exhibited breast lesions that were not categorized as BI-RADS 4 following MRI assessment. (2) The subjects underwent biopsy or surgical resection prior to the MRI examination. (3) The availability of relevant data was limited or incomplete despite attempts to contact the authors.

### Literature screening and data extraction

Two reviewers used EndNote (v20.0) to import and removed duplicate records from the retrieved literature. The reviewers then conducted an initial screening of titles and abstracts to identify original research data that met the criteria. The selected data was then downloaded for further in-depth reading and analysis. When the included literature had limited or incomplete data, two reviewers contacted the original authors via email to request complete data. If obtaining complete data was not possible, the paper was excluded from the analysis. The final set of literature included in this meta-analysis was determined based on the predefined inclusion and exclusion criteria.

Prior to data extraction, two reviewers developed a standardized extraction form that included various parameters such as article title, author names, publication year, author’s country, study design, MRI equipment manufacturer, MRI magnetic field strength, feature screening algorithm, radiomics algorithm, contrast agent type, and ratio of the test set to the training set. The reviewers extracted the values of true positive (TP), false positive (FP), true negative (TN), and false negative (FN) from the data to generate a 2 × 2 contingency table. In cases where multiple radiomics models were reported in a single study, the model with the highest diagnostic accuracy was selected for meta-analysis. Any disagreements between the two reviewers were resolved through discussion until a consensus was reached.

### Data quality assessment

The selected studies will undergo quality assessment using the Radiomics Quality Score (RQS) scale, originally proposed by Lambin et al. in 2017. This scale comprises 16 items that evaluate various aspects, including image acquisition, radiomics feature extraction, data modeling, model validation, and data sharing. Each item is assigned a specific score based on the extent to which the research fulfills the criteria. The score range is from  – 8 to 36 (Lambin et al. 2017), which is subsequently converted into a percentage score ranging from 0 to 100%. Scores from  – 8 to 0 are considered as 0%, while a score of 36 is considered as 100%. To ensure a comprehensive assessment of study quality in this meta-analysis, the Quality Assessment of Diagnostic Accuracy Studies (QUADAS-2) scale is also employed to evaluate the risk of bias in the included studies (Whiting et al. 2011).

### Statistical analysis

In this study, Meta-Disc 1.4 software was utilized to conduct a heterogeneity test, and the threshold effect was assessed by calculating Spearman’s correlation coefficient. A threshold effect was considered present when the calculated P value was less than 0.05 (Devillé et al. 2002). The heterogeneity among the included studies was evaluated using the Cochran Q test and the I^2^ statistic. Statistical significance was determined when the *P* value was less than 0.05, and a value of I^2^ greater than or equal to 50% indicated moderate to high heterogeneity across the studies (DerSimonian and Laird 1986).

This study calculated sensitivity, specificity, positive likelihood ratio (PLR), negative likelihood ratio (NLR), and diagnostic odds ratio (DOR) with 95% confidence intervals (95% CI). Summary receiver operating characteristic (SROC) analysis generated the area under the curve (AUC). A sensitivity analysis excluded two studies that only analyzed the test set. Publication bias was assessed using a Deeks funnel plot, with a slope coefficient’s *P* value below 0.10 indicating significant publication bias (Deeks et al. 2005). Statistical analyses utilized STATA 16.0’s ‘midas’ module.

## Results

**Fig.1:**
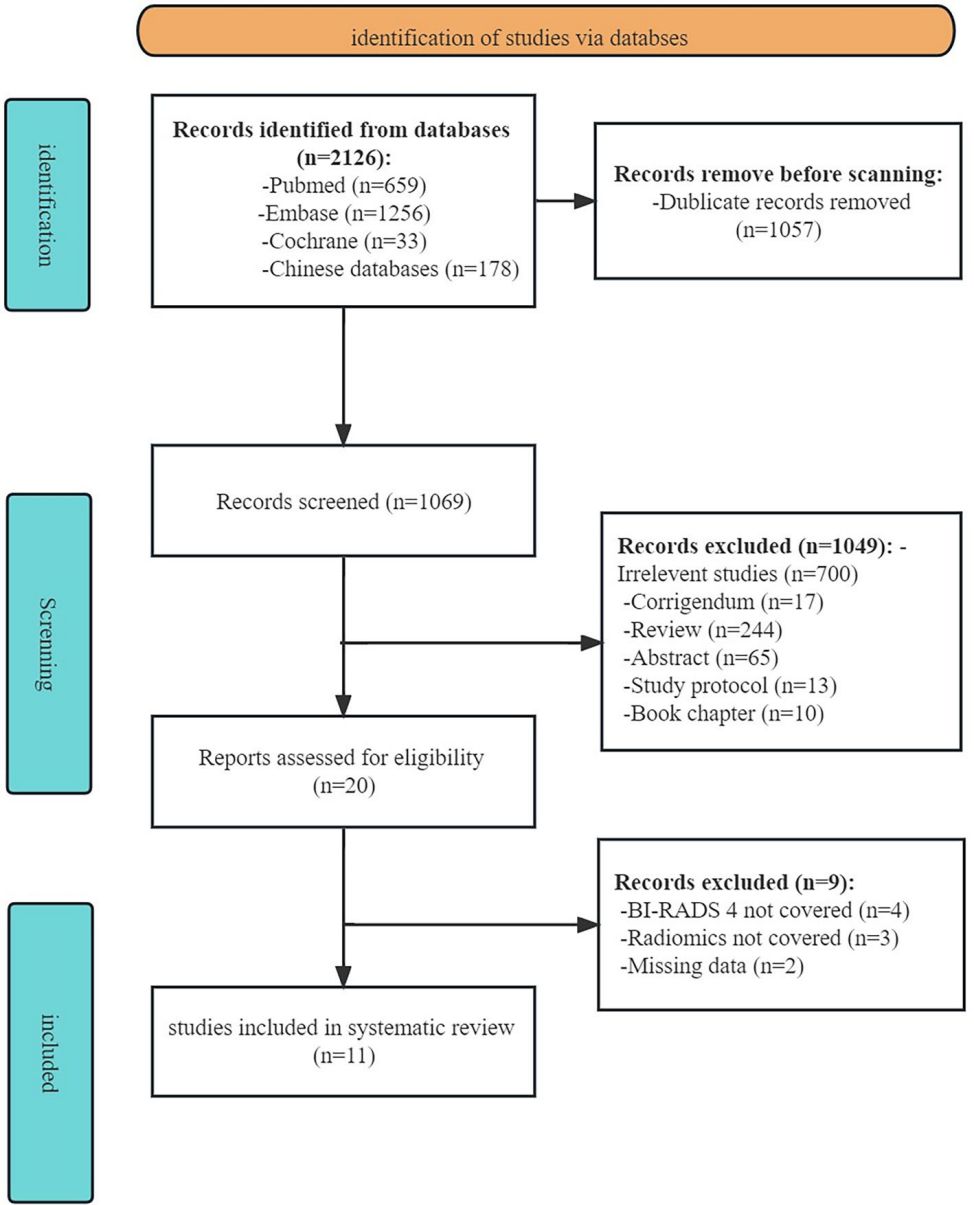
PRISMA flow diagram of Study Selection

A comprehensive search across four public databases initially identified 2,012 records. After removing 943 duplicate documents, a meticulous evaluation of titles, abstracts, and full-text articles led to the exclusion of 1,049 studies, encompassing reviews, letters, conference reports, and other publications unrelated to the subject matter. Subsequent re-screening of the remaining 20 literature sources was performed based on predefined inclusion and exclusion criteria. Consequently, 9 articles were found ineligible and excluded from the analysis. Finally, 11 articles meeting the defined criteria were included in the meta-analysis (Zhang et al. 2022). The PRISMA flowchart below visually illustrates the study selection process (Fig. 1).

### Study overview

This meta-analysis analyzed 11 studies (Ellmann et al. 2020; Hu et al. 2017; Bickelhaupt et al. 2018; Yin et al. 2022; Hu et al. 2023; Zhao et al. 2021; Zhou et al. 2021; Bakker et al. 2019; Liu et al. 2019; Rao et al. 2016; Thibault et al. 2013a) published between December 2017 and March 2023, which collectively involved 2208 patients with 2230 lesions identified as BI-RADS 4. The omics method was used to evaluate 1915 lesions in 1893 patients, reflecting the practical application of imaging techniques. Among the included studies, 8 were from China, while the remaining 3 were conducted in Austria, Germany, and the United States. Machine learning algorithms were utilized in 5 studies, deep learning algorithms in 3 studies, and the algorithms used in the remaining 3 studies were not explicitly specified. The table below presents a detailed summary of the radiomics and associated imaging data extracted from the 11 studies included in the analysis (Table 1).

**Table 1:** Characteristics of enrolled studies. NA means not available

Research	Region	MRI equipment	Magnetic field strength	Radiomics algorithms	MRI sequence	Number of patients	Number of lesions	Malignant	Benign
Zhang et al. (2022)	China	NA	3.0 T	Machine learning	T_2_WI + DWI + DCE	216	216	166	50
Hao et al. (2020)	China	Siemens	3.0 T	Machine learning	T_1_WI + DCE + T_2_WI	178	178	97	81
Lyu et al. (2023)	China	GE	3.0 T	Deep learning	DISCO-10 + DISCO-15	173	182	87	95
Naranjo et al. (2021)	USA	Siemens/GE	3.0 T	Machine learning	DCE + DWI	93	104	46	58
Ellmann.et al. (2020)	Austria	Siemens	3.0 T/1.5 T	Machine learning	T_1_WI + T_2_WI + DWI + DCE	100	100	76	24
Hu.et al. (2017)	China	Siemens	3.0 T	NA	T_1_WI + T_2_WI + DWI + DCE	25	25	15	10
Bickelhaupt. et al. (2018)	Germany	Siemens/Philips	1.5 T	NA	DWI + T_2_WI	103	103	38	65
Yin et al. (2022)	China	Siemens	3.0 T	Deep learning	T_1_WI + T_2_WI + DCE	67	67	27	40
Hu et al. (2023)	China	GE	3.0 T	NA	T_1_WI + T_2_WI + DWI + DCE	522	522	501	21
Zhao et al. (2021)	China	GE	3.0 T	Machine learning	T_1_WI + DCE + T_2_WI	266	268	194	74
Zhou et al. (2021)	China	GE	3.0 T	Deep learning	DCE	150	150	104	46

*MRI* magnetic resonance imaging, *T1WI* T1-weighted image, *T2WI* T2-weighted image, *DCE* dynamic contrast-enhanced, *DWI* diffusion-weighted imaging, *DISCO* differential subsampling with cartesian ordering

### RQS and QUADAS-2 assessment

**Fig. 2:**
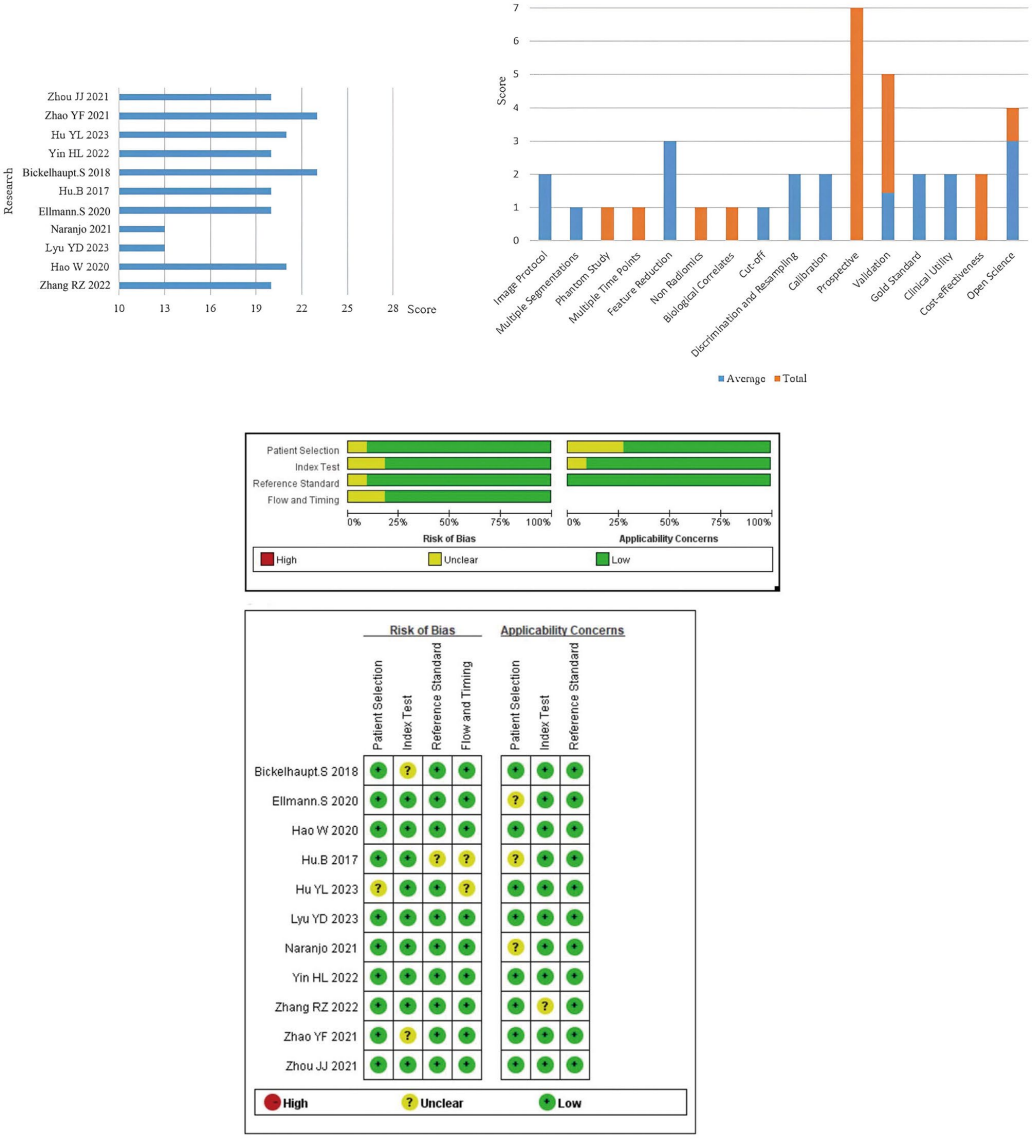
The RQS scores and QUADAS-2 scores for 11 studies

The included studies exhibited a median RQS score of 20, ranging from 13 to 23 points, representing 55% of the total RQS score, which ranged from 36 to 63%. Notably, one study attained the highest score of 23 points (63%) (Bickelhaupt et al. 2018), while two studies received the lowest score of 13 points (36%) (Bickelhaupt et al. 2018; Yin et al. 2022) due to their failure to conduct internal and external validation. In terms of individual item assessment, all studies met the criteria for eight indicators: image protocol, multiple segmentation, feature reduction, cutoff determination, calibration, discrimination and resampling, gold standard reference, and clinical utility. However, the indicators related to the phantom study, prospective study, cost-effectiveness, biological correlates, nonradiomics factors, and multiple time points received a score of 0, as none of the included studies addressed these specific aspects. To ensure a comprehensive assessment of study quality in this meta-analysis, the QUADAS-2 scale is also employed to evaluate the risk of bias in the included studies (Whiting et al. 2011). According to the QUADAS-2 assessment, most of the studies had low risk of bias and limited applicability concerns, indicating high-quality data. A comprehensive summary of the RQS scores and a detailed summary of the risk of bias and applicability concerns for all the studies included can be found in Fig. 2.

### MRI radiomics-based diagnosis of BI-RADS 4 benign and malignant breast lesions

**Fig. 3:**
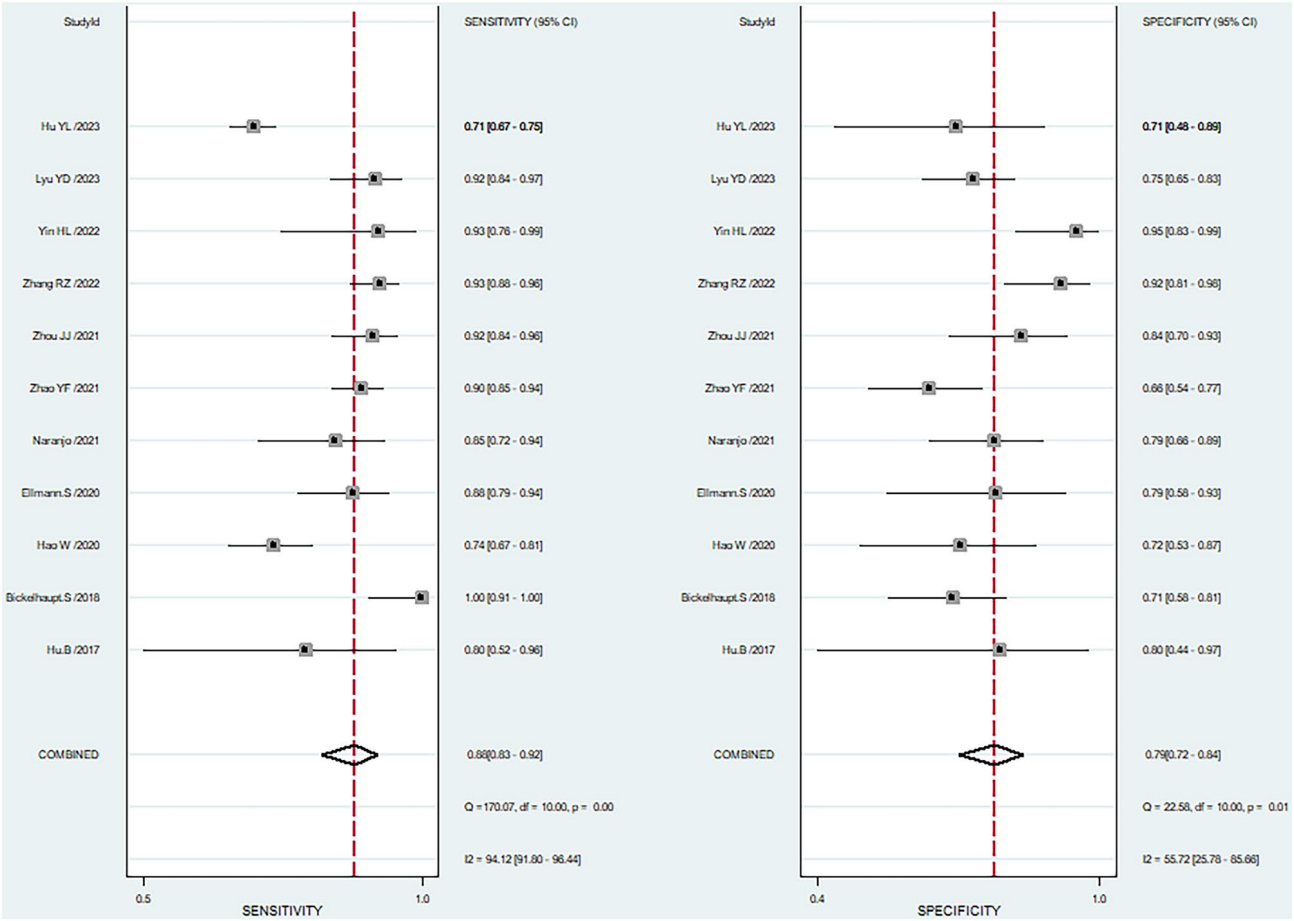
The Forest plot. A meta-analysis of 11 studies evaluated the diagnostic accuracy of radiomics in distinguishing between BI-RADS 4 benign and malignant breast lesions. The results were summarized in this plot displaying pooled estimates and 95% confidence intervals

**Fig. 4:**
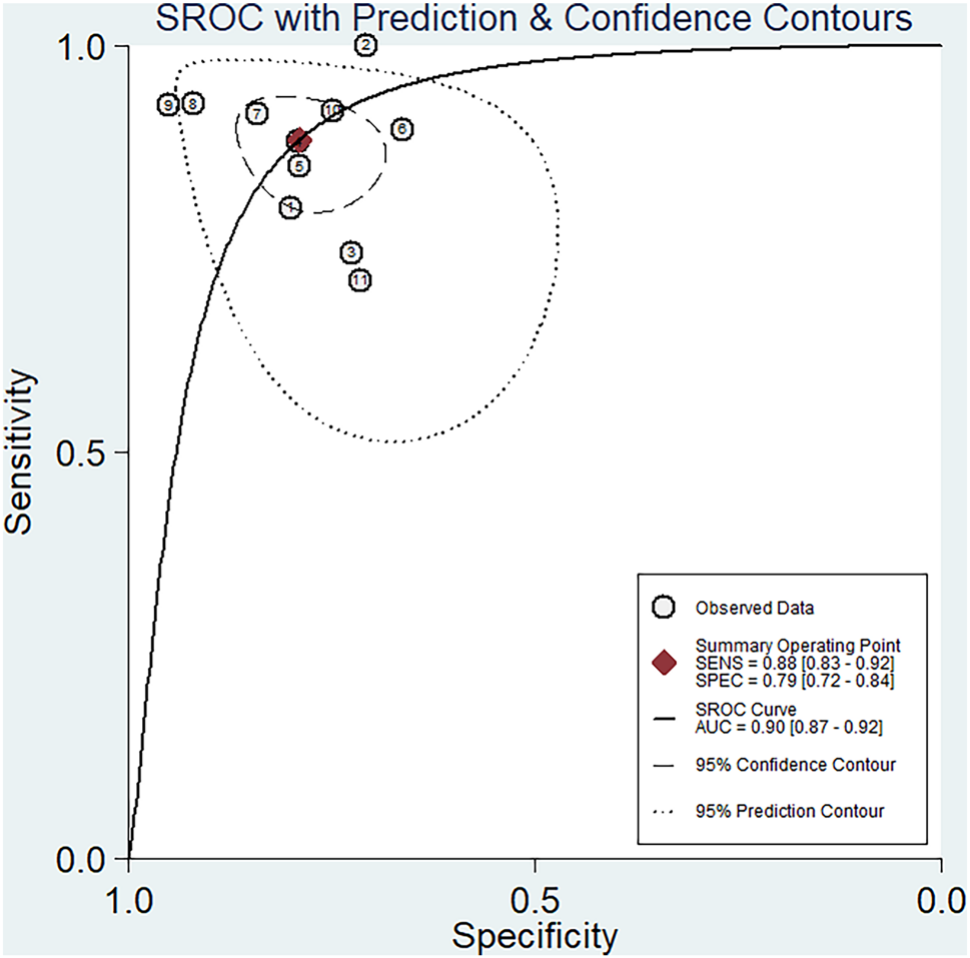
The SROC diagram. It was created to demonstrate the diagnostic performance of radiomics in differentiating benign and malignant breast lesions classified as BI-RADS 4. The numbers within the circles correspond to the order of articles listed in Table

The threshold analysis revealed no threshold effect between MRI radiomics-based diagnosis of benign and malignant breast lesions in BI-RADS 4, with a correlation coefficient of *r* =  – 0.246 (*p* = 0.466). The study included 1893 patients with a total of 1915 lesions, out of which 1351 were malignant and 570 were benign. The combined findings are presented in Fig. 3, illustrating a forest plot. The combined results are visually presented in Fig. 4, which displays a SROC diagram. The combined sensitivity was 0.88 (95% CI 0.83–0.92, *I*^2^ = 76.44%), specificity was 0.79 (95% CI 0.72–0.84, *I*^2^ = 55.72%), PLR was 4.2 (95% CI 3.1–5.7), NLR was 0.15 (95% CI 0.10–0.22), DOR was 29.0 (95%CI 15–55), and AUC based on SROC curve was 0.90 (95% CI 0.87–0.92), indicati1ng a high diagnostic value.

### Subgroup and sensitivity analyses

Subgroup analyses were conducted on sixteen subgroups across eight distinct conditions. These conditions included race (Asian and non-Asian), training set population to test set population ratio (7/3 and non-7/3), feature selection algorithm (LASSO and non-LASSO), contrast agent (gadobenate dimeglumine and non-gadobenate dimeglumine), tumor segmentation (manual and automatic), MRI equipment (Siemens and GE), magnetic field strength (3.0 T and 1.5 T), and radiomics algorithms (machine learning and deep learning).

**Fig. 5:**
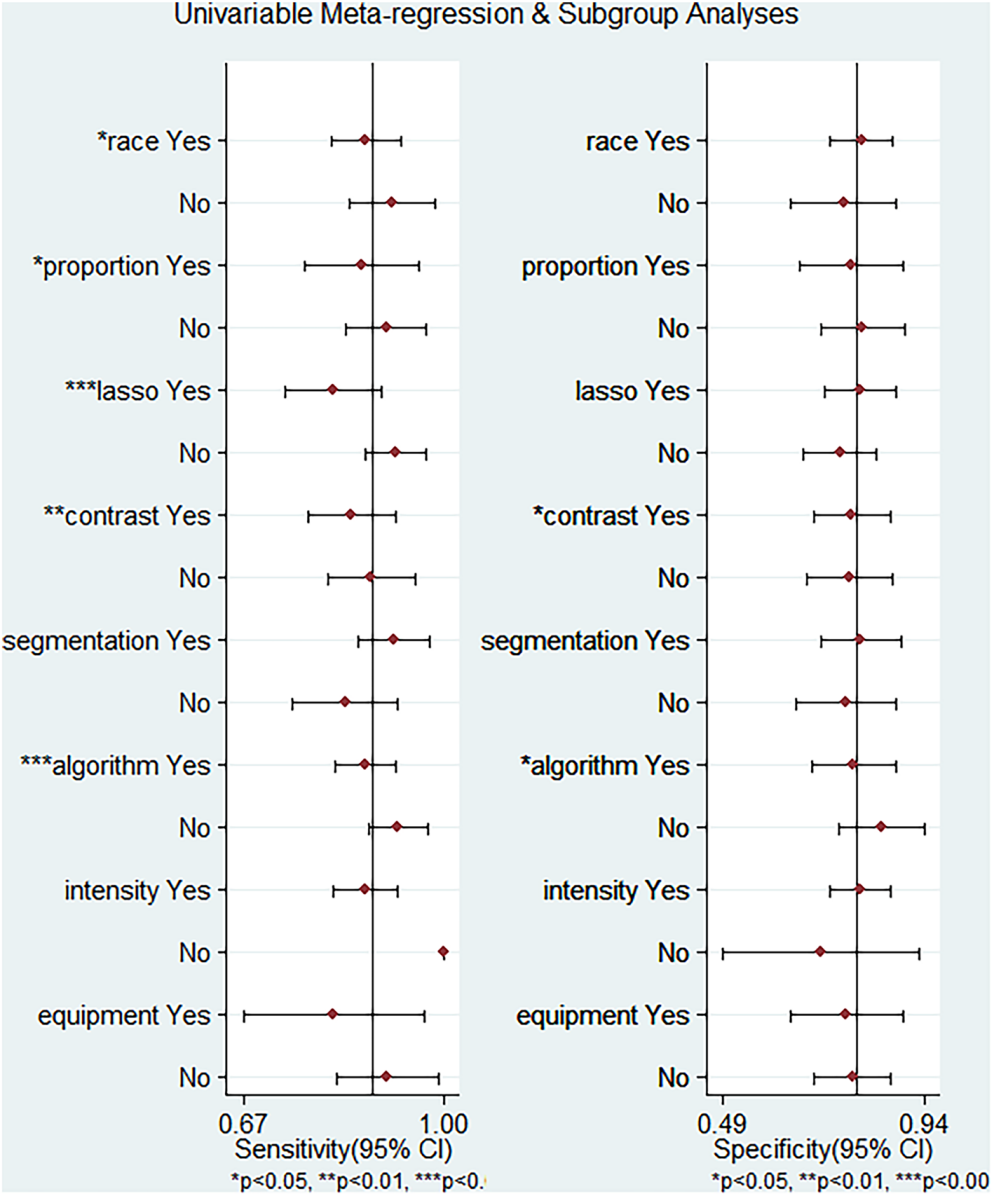
The Heterogeneity analysis

Regression analysis revealed significant sources of heterogeneity in all conditions, with the exception of race. Detailed illustrations of these findings are presented in the accompanying figure and table below (Fig. 5; Table 2).

**Table 2:** Subgroup Analyses

Subgroups	Group	Research number	Sensitivity (95%CI)	Specificity (95%CI)
Race	Asian	8	0.87 (0.82–0.93)	0.80 (0.73–0.87)
	Non-Asian	3	0.92 (0.84–0.99)	0.76 (0.64–0.88)
Raining set population to test set population ratio	7/3	4	0.87 (0.77–0.96)	0.78 (0.66–0.90)
	Non-7/3	5	0.91 (0.84–0.97)	0.80 (0.71–0.90)
Feature selection algorithm	LASSO	4	0.82 (0.74–0.90)	0.80 (0.72–0.88)
	Non-LASSO	4	0.92 (0.86–0.98)	0.75 (0.67–0.84)
Contrast agent	Gadobenate dimeglumine	5	0.85 (0.78–0.92)	0.7 8(0.69–0.87)
	Non-gadobenate dimeglumine	4	0.88 (0.81–0.95)	0.77 (0.68–0.67)
Tumor segmentation	Manual	5	0.92 (0.86–0.98)	0.80 (0.71–0.89)
	Automatic	4	0.84 (0.75–0.93)	0.77 (0.65–0.88)
Radiomics algorithms	Machine learning	5	0.87 (0.82–0.92)	0.80 (0.73–0.87)
	Deep learning	3	0.93 (0.88–0.97)	0.85 (0.75–0.94)
Magnetic field strength	3.0T	9	0.87 (0.82–0.92)	0.80 (0.73–0.87)
	1.5T	1	1 (1.00–1.00)	0.72 (0.49–0.93)
MRI equipment	Siemens	3	0.82 (0.67–0.97)	0.77 (0.69–0.89)
	GE	4	0.91 (0.82–0.99)	0.78 (0.69–0.87)

*MRI* magnetic resonance imaging, *LASSO* Least Absolute Shrinkage and Selection Operator

In this study, a sensitivity analysis was conducted by excluding studies that only performed radiomics analysis on the test set data (Zhao et al. 2021; Bakker et al. 2019). The data was then re-analyzed, and it was found that there were no significant changes in sensitivity (0.88 vs 0.88) and specificity (0.79 vs 0.77). In addition, an exclusion analysis was performed to investigate the potential impact of database origin on heterogeneity. Specifically, one Chinese literature article published in databases (Hu et al. 2017) was removed, and the remaining 10 English literature articles were re-evaluated. The study found that there were no significant changes in sensitivity (0.88 vs 0.89) and specificity (0.79 vs 0.79). The consistent results reinforce the robustness and reliability of the findings, providing a solid foundation for the stability and credibility of the conclusions drawn in this study.

### The Publication bias and the influence of diagnostic results on disease prevalence probability

**Fig. 6:**
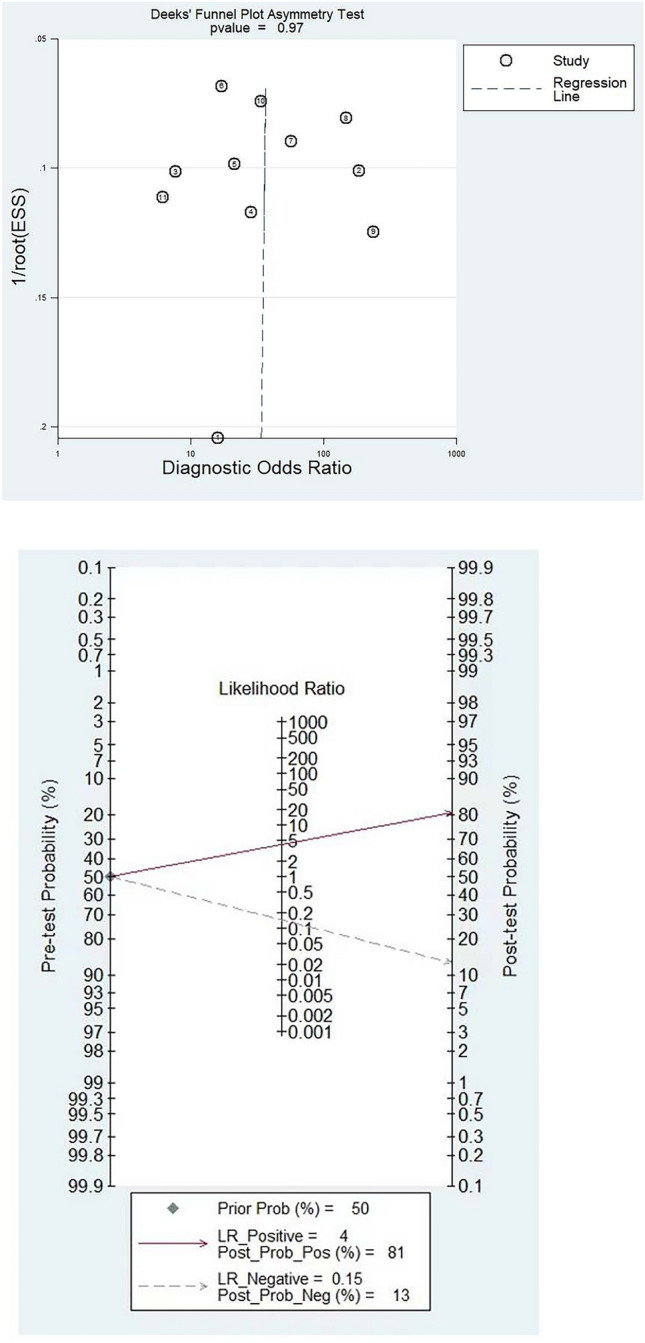
The Deeks funnel plot and the Fagan diagram. The former assessed MRI radiomics in differentiating BI-RADS 4 benign and malignant breast lesions. The plot’s serial numbers correspond to reference order. The latter evaluated MRI radiomics’ diagnostic efficacy for BI-RADS 4 lesions

The Deeks funnel chart showed no evidence of publication bias, while the Fagan diagram illustrates that the probability of correctly predicting BI-RADS 4 benign and malignant breast lesions before using MRI radiomics is initially 50%. However, when MRI radiomics is used in the prediction process, the probability of predicting positive cases increases significantly to 81%, while the probability of predicting negative cases decreases to 13%. These results indicate that MRI radiomics improves the diagnostic accuracy of BI-RADS 4 benign and malignant breast lesions, highlighting the usefulness of this technique in clinical practice. Detailed illustrations of these findings are presented in the accompanying figure below (Fig. 6).

## Discussion

Breast MRI shows promise as a modality for breast screening due to its superior soft tissue resolution and lack of radiation exposure. Studies have demonstrated its significant superiority over mammography and ultrasonography in terms of early breast cancer diagnosis and local staging (Thibault et al. 2013b; Hall-Beyer 2017). The BI-RADS (Rao et al. 2016) scoring system is a standardized way to assess the imaging characteristics of breast tumors and provide an approximate evaluation of lesion malignancy. The current classification system for breast cancer does not adequately account for the variations among tumors, resulting in the diverse biological behaviors observed. The NCCN guidelines recognize that BI-RADS 4 breast lesions have a malignancy probability ranging from 2 to 95%. However, the positive predictive value falls between 25.7% and 59.2% in reality (Vandenbroucke et al. 2014; Altman et al. 2001; Willinek and Schild 2008). Artificial intelligence has become increasingly prominent in the exploration and application of medical image data, as it overcomes the limitations associated with visual interpretation of tumor images (Lambin et al. 2017). It provides a noninvasive approach for predicting malignancy in breast lesions based on imaging data, offering diagnosticians and oncologists a reliable quantitative assessment tool.

This meta-analysis analyzed 11 studies that evaluated the effectiveness of MRI radiomics in detecting benign and malignant breast lesions classified as BI-RADS 4. The study included 1893 patients who were diagnosed with BI-RADS 4 breast lesions, which accounted for a total of 1915 lesions. The analysis results demonstrated that MRI radiomics exhibited high sensitivity and specificity in diagnosing BI-RADS 4 benign and malignant breast lesions. These findings highlight the potential of combining radiomics with MRI for the diagnosis of BI-RADS 4 lesions. Specifically, the study’s analysis indicated that the probability of accurately identifying true positive patients was 4.2 times greater than that of false positive patients (combined PLR = 4.2), resulting in a significant increase in the likelihood of detecting positive cases, while the probability of true negative patients was approximately 6.6 times higher than that of false negative patients (combined NLR = 0.15), indicating a substantial reduction in the likelihood of incorrectly identifying negative cases. These findings indicate that MRI-based radiomics exhibits substantial diagnostic efficacy in distinguishing between BI-RADS 4 benign and malignant breast lesions, underscoring its significant practical utility and wide-ranging applications.

The meta-analysis identified heterogeneity in the study, which could be attributed to various factors such as the ratio of individuals in the training set to the test set, contrast agents, tumor segmentation methods, feature selection algorithms, MRI equipment manufacturers, magnetic field strengths, and radiomics algorithms. It was concluded that proper allocation of individuals between the training and test sets is essential for the outcomes of machine learning and deep learning models (Song et al. 2020; Sedgwick 2013). Therefore, it is recommended to carefully determine an appropriate ratio to ensure reliable and robust results.

Notably, the unexpected finding of higher sensitivity in 1.5 T MRI as compared to 3.0 T MRI challenges the conventional understanding that higher magnetic fields enhance image resolution and diagnostic accuracy (Willinek and Schild 2008). However, this meta-analysis only included one study on 1.5 T MRI, indicating the necessity for further investigations with more 1.5 T MRI studies to confirm this observation.

The study found that manual segmentation has better sensitivity and specificity than automatic segmentation, potentially due to limitations in current automatic segmentation algorithms. Manual segmentation enables precise control through manual intervention, resulting in higher accuracy and comprehensiveness. Deep learning has exhibited higher sensitivity and specificity than machine learning in radiomics algorithms, but this observation is based on only three studies. Hence, further research is required to ensure the reliability and generalizability of these findings.

Subgroup analysis indicated that studies utilizing GE MRI equipment showed higher diagnostic performance compared to those using Siemens equipment, suggesting the impact of different MRI devices on diagnostic accuracy. Therefore, a prospective study is needed to compare the diagnostic performance of BI-RADS 4 breast benign and malignant lesions using these specific MRI devices based on MRI radiomics.

LASSO (Least Absolute Shrinkage and Selection Operator) is a commonly used feature selection technique in high-dimensional data analysis and regression (Ranstam et al. 2018). Previous research has shown that LASSO and logistic regression are commonly used in radiomics-related studies (Song et al. 2020). Our investigation found that LASSO has significant heterogeneity when compared with non-LASSO methods such as support vector machines, highlighting the need for further examination of the factors contributing to this heterogeneity. When using the LASSO method in future studies on diagnosing BI-RADS 4 breast lesions using MRI radiomics, it is important to carefully select an appropriate feature screening algorithm that aligns with the dataset’s characteristics to optimize the balance between sensitivity and specificity. This will enhance diagnostic accuracy and reliability.

In addition, it is important to consider the potential biases introduced by different contrast agents when using MRI radiomics for the diagnosis of BI-RADS 4 breast lesions. This requires an understanding of how the chemical structure of the contrast agent may impact the results. By taking these factors into account, we can improve our understanding and control of potential influencing factors, ultimately leading to more accurate and reliable outcomes.

To account for potential heterogeneity from the database source, we excluded the Chinese literature (Hu et al. 2017) from the 11 studies that were included. We then conducted a reanalysis using the remaining 10 studies that were in English. Our findings showed that there were no significant changes in sensitivity (0.88 vs. 0.89) and the degree of specificity (0.79 vs. 0.79).

The results of our study demonstrate stability and reliability. Nonetheless, it is crucial to recognize certain limitations. Firstly, it is important to note that all 11 studies analyzed were retrospective in nature, which means they lacked the rigorous data collection and control measures typically associated with prospective studies. Secondly, our meta-analysis revealed substantial heterogeneity in sensitivity (95% CI 0.83–0.92, *I*^2^ = 76.44%) and specificity (95% CI 0.72–0.84, *I*^2^ = 55.72%) due to variations in the statistical methods employed across different studies, encompassing model selection and effect size estimation methods. Despite this heterogeneity, the results of our study demonstrate stability and reliability. However, the literature reviewed in our study primarily focused on the effectiveness of MRI radiomics in diagnosing benign and malignant BI-RADS 4 breast lesions, but did not extensively evaluate specific subtypes within these categories. Future meta-analyses should consider including more studies that specifically target the assessment of subtypes within the benign and malignant BI-RADS 4 classifications. This would improve the comprehensiveness of the analysis and provide more valuable insights into the diagnostic capabilities of MRI radiomics for distinguishing between these subtypes.

Notably, in our study, 9 out of the 11 included studies utilized contrast-enhanced sequences, specifically Dynamic Contrast-Enhanced (DCE) sequences, for breast scanning. However, the role of other sequences in diagnosing BI-RADS 4 breast cancer, such as diffusion-weighted imaging (DWI), has been overlooked. This is a nonenhanced MRI technique that rapidly evaluates tissue characteristics by measuring the movement of water molecules within the tissues. In the context of breast cancer, restricted diffusion of water molecules is observed in comparison to normal breast tissue, resulting in a higher signal on DWI (Partridge et al. 2017). The recent studies have demonstrated the effectiveness of DWI-based radiomics in assessing the heterogeneity of breast tumors, providing valuable guidance for treatment decisions and noninvasive tumor monitoring (Leithner et al. 2020).Consequently, future research is expected to explore the potential of DWI radiomics in the evaluation of BI-RADS 4 benign and malignant breast lesions, presenting an exciting avenue for further investigation.

## Conclusion

In conclusion, MRI-based radiomics has high diagnostic accuracy for BI-RADS 4 breast lesions. Further validation in large-scale studies will enhance patient outcomes by providing accurate diagnoses and guiding effective treatments.

## Data Availability

The datasets analyzed during the current study are available in the public databases PubMed, Embase, Cochrane Library, and Sinomed. The data from the 11 studies included can be traced and accessed via the DOIs provided in the references section of our manuscript.
